# Plastic Responses Contribute to Explaining Altitudinal and Temporal Variation in Potential Flower Longevity in High Andean *Rhodolirion montanum*

**DOI:** 10.1371/journal.pone.0166350

**Published:** 2016-11-18

**Authors:** Diego Andrés Pacheco, Leah S. Dudley, Josefina Cabezas, Lohengrin A. Cavieres, Mary T. K. Arroyo

**Affiliations:** 1 Instituto de Ecología y Biodiversidad, Santiago, Chile; 2 Departamento de Ciencias Ecológicas, Facultad de Ciencias, Universidad de Chile, Santiago, Chile; 3 Biology Department, University of Wisconsin-Stout, Menomonie, Wisconsin, United States of America; 4 Departamento de Botánica, Facultad de Ciencias Naturales y Oceanográficas, Universidad de Concepción, Concepción, Chile; Indian Institute of Science, INDIA

## Abstract

The tendency for flower longevity to increase with altitude is believed by many alpine ecologists to play an important role in compensating for low pollination rates at high altitudes due to cold and variable weather conditions. However, current studies documenting an altitudinal increase in flower longevity in the alpine habitat derive principally from studies on open-pollinated flowers where lower pollinator visitation rates at higher altitudes will tend to lead to flower senescence later in the life-span of a flower in comparison with lower altitudes, and thus could confound the real altitudinal pattern in a species´ potential flower longevity. In a two-year study we tested the hypothesis that a plastic effect of temperature on flower longevity could contribute to an altitudinal increase in potential flower longevity measured in pollinator-excluded flowers in high Andean *Rhodolirium montanum* Phil. (Amaryllidaceae). Using supplemental warming we investigated whether temperature around flowers plastically affects potential flower longevity. We determined tightly temperature-controlled potential flower longevity and flower height for natural populations on three alpine sites spread over an altitudinal transect from 2350 and 3075 m a.s.l. An experimental increase of 3.1°C around flowers significantly decreased flower longevity indicating a plastic response of flowers to temperature. Flower height in natural populations decreased significantly with altitude. Although temperature negatively affects flower longevity under experimental conditions, we found no evidence that temperature around flowers explains site variation in flower longevity over the altitudinal gradient. In a wetter year, despite a 3.5°C temperature difference around flowers at the extremes of the altitudinal range, flower longevity showed no increase with altitude. However, in a drier year, flower longevity increased significantly with altitude. The emerging picture suggests an increase in flower longevity along the altitudinal gradient is less common for potential flower longevity than for open-pollination flower longevity. Independently of any selection that may occur on potential longevity, plastic responses of flowers to environmental conditions are likely to contribute to altitudinal variation in flower longevity, especially in dry alpine areas. Such plastic responses could push flowers of alpine species towards shorter life-lengths under climate change, with uncertain consequences for successful pollination and plant fitness in a warming world.

## Introduction

Altitudinal gradients show two striking global trends related to plant reproduction. First, pollination rates decline with decreasing temperatures and more unpredictable weather conditions at higher altitudes [1–11]. Second, flower longevity often increases with altitude [5,6,12–17], there being outstanding cases of nival plants with extremely long-lived stigmas [18,19]. The above two trends are considered to be functionally linked, with longer-lived flowers compensating for lower pollination rates at higher elevations [5,6,13]. Flowers that have the capacity to stay open for long periods of time in general allow more time for pollinator visits to accumulate [20,21] and thus can increase the probability of successful pollination when pollinator availability reaches a critical threshold. Nevertheless, physiological costs associated with transpiration, respiration and nectar production in flowers can drain resources for later fruit and seed set [22,23] and thus place limits on flower longevity, such that pollinator availability in natural populations is not the only factor molding flower longevity.

A broad meta-analysis found no difference in pollen-limitation levels for alpine and lowland species ([24], see also [25]); Wagner et al. [26] recently established that nival flowers in the Tyrolean Central Alps are not pollinator-visitor limited. This could be because alpine species possess compensatory traits that allow them to deal with pollinator scarcity thereby decreasing pollen limitation [27–30]. One such trait is potential flower longevity, the time a flower is able to remain open be and available to be pollinated. This trait has been shown to be associated with low visitation rates due to high co-flowering diversity [23] and therefore could also be associated with low visitation rates at higher altitudes. Flower longevity has been shown to increase with altitude; however the great majority of studies that show an altitudinal increase in flower longevity in the alpine (see [12]) have relied on measurements of flower longevity in open-pollinated flowers. As mentioned earlier, pollination rates tend to be lower at higher altitudes in the alpine [1–11]. This, taken together with the fact that pollination tends to hasten flower senescence [31–33], leading to a negative correlation between open-pollinated flower longevity and pollination rates (e.g. [34,35]), signifies that an altitudinal increase in the longevity of open-pollinated flowers in the alpine will not necessarily reflect a real altitudinal difference in a species´ potential flower longevity. Potential flower longevity, by definition, refers to flower longevity measured in pollinator-excluded flowers and refers to the maximum amount of time a flower can remain open. This raises the question as to whether potential flower longevity is also expected to increase with altitude, and if so how this might be explained. To date the few available studies on potential flower longevity over the altitudinal gradient in the alpine show inconsistent trends [5,15,36,37].

High altitude plants, in addition to the global altitudinal temperature decrease, must contend with significant small-scale spatial temperature variation in the soil [38], at the soil surface [39,40] and close to the ground [41,42]. This local temperature variation arises from complex interplay between topography, incoming radiation and atmospheric circulation and can be associated with notable differences in pollinator composition and flower visitation rates [41,43]. How alpine flowers respond to such local temperature variation could provide an important clue on what may be expected in relation to potential flower longevity over the altitudinal gradient. Arroyo et al. [44] recently reported a surprisingly large amount of local temperature-driven plasticity in potential flower longevity in high altitude *Oxalis compacta*, which had a positive effect on seed set under low temperatures. If differences in potential flower longevity can arise as a plastic response to local temperature variation, variation in flower longevity of the same nature could be manifest over the larger-scale altitudinal gradient where temperature declines by a global average of around 6.0°C per 1000 m increase in altitude [45]. Additional support for the notion that temperature-driven plasticity could mold flower longevity over the altitudinal gradient comes from more prolonged flower longevity in individuals that flower under cooler temperatures early in the flowering season [46,47] and from controlled laboratory experiments [48–51]. These trends are not unexpected; flower respiration rates are known to increase with flower tissue temperature under laboratory conditions [52] and lower respiration rates associated with cooler temperatures are known to prolong flower lifespans in cut flowers [53,54]. In spite of its appealing simplicity, that temperature-driven plasticity could contribute to (although not necessarily explain all) altitudinal variation in potential flower longevity over the alpine gradient has not been tested. A test of this hypothesis in natural populations requires three conditions: 1) The source of external pollination on flower longevity must be eliminated; 2) Flower longevity measurements must be representative of the entire flowering season at each altitude so as to eliminate spurious differences in flower longevity arising from seasonal displacement of flowering and weather variation at different altitudes; 3) Given that temperature increases rapidly close to the ground in the alpine [42,55], temperature around flowers should be measured at the height above ground at which flowers are disposed. No study on potential flower longevity in the alpine, as far as we can tell, complies completely with all these conditions. Finally, prior experimental evidence of a plastic response of flowers to temperature (4) is desirable.

Here, in a two-year study incorporating the above four considerations, we investigate flower longevity measured in pollinator-excluded flowers over the altitudinal gradient in the high Andean *Rhodolirium montanum* (Amaryllidaceae). We focused principally on three questions: 1) Does temperature affect flower longevity under experimental conditions? 2) Does potential flower longevity increase over the altitudinal gradient in *R*. *montanum*? 2) Does temperature-driven plasticity contribute to any altitudinal variation in potential flower longevity in this species? An experimental increase in potential flower longevity is expected given that flower development will be faster under warmer temperatures. If higher temperatures reduce flower longevity experimentally, given that temperature declines with altitude, an increase in potential flower longevity is expected with increasing altitude. However, although temperature might affect flower longevity experimentally, other factors could override the effect of temperature in natural population and be more important in molding the altitudinal trend. Even if evidence is found for a contribution of plasticity related to temperature or some other abiotic factor to altitudinal variation in flower longevity, this of course does not negate the possibility that selection on flower longevity has also occurred and contributes together with a plasticity in molding the altitudinal trends. That is a temperature effect and selection could be combined.

## Materials and Methods

### Study species

**Fig 1:**
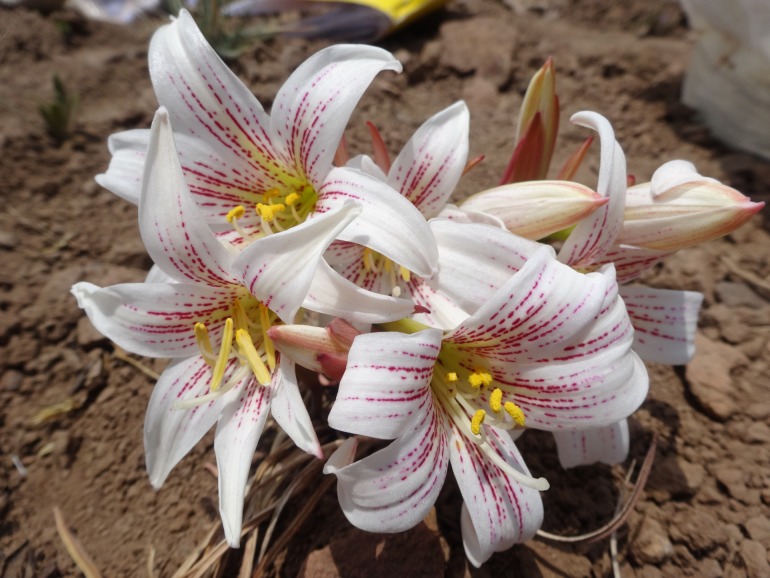
Flowers of *Rhodolirium montanum*.

*Rhodolirium montanum* Phil (syn: *Rhodophiala rhodolirion* (Baker) Traub.) (Amaryllidaceae) (Fig 1) is a showy bulbous geophyte that occurs from the level of the subalpine zone above the *Kageneckia angustifolia* tree line into the high alpine zone (2200–3100 m a.s.l.) in the central Chilean Andes (33°S). Small outlying populations can be found at 1800 m a.s.l. and 3250 m a.s.l. Leaves appear as the snow melts in spring to wither in early summer before the flowers appear [56]. Each large bulb produces an inflorescence with a single (very rarely two) flower supported by a robust peduncle. Flowering occurs from late December to early-February, depending on year. In the study area, *R*. *montanum* occurs as isolated individuals, or as clusters of asexually produced bulbs (genets) connected by a thin thread of cells to the basal plate. Asexual bulb replication is more prevalent in the upper part of the altitudinal range. *Rhodolirium montanum* is partially self-compatible, strongly herkogamous, non-autogamous, and bee-pollinated [57]. The showy, pendant flowers are reported to remain open for around one week [57] (Fig 1). Tepals of mature flower buds burst in the early evening or early the next morning. Stigmas are receptive in first day flowers, with liquid on the stigma discernable by four days after anthesis on many stigmas; pollen sheds on the first day of anthesis [57]. In the study area community-level pollination rates decline by 50% from the subalpine to the high alpine [1]. Preliminary work in the same general area as the present study failed to detect pollen limitation in *R*. *montanum* [57]. However, it would be worthwhile revisiting this conclusion as sample sizes in this early study were severely reduced by animal damage.

### Study sites

Work took place in the Cordillera de Los Andes in central Chile (33° S) to the east of Santiago (33°S) between Farellones and Valle Nevado over three austral summer seasons (2010–2011, 2013–2014 and 2014–2015). The area has an alpine climate with a strong mediterranean-climate influence [56,58]. Annual precipitation in the general study area descends from > 900 mm at 3200 m to < 500 m at 2500 and falls mostly as snow; rainfall is scarce over the summer months [59]. We worked on three sites separated by approximately equal altitudes over *R*. *montanum*´s core altitudinal range (Table 1). The final selection of sites obeyed the need for large populations with many flowers. The LOW site was at 2350 m a.s.l., the MID site at 2650 m a.s.l. and the HIGH site at 3075 m a.s.l. Importantly, flowering at different altitudes in *R*. *montanum* is strongly overlapping at all altitudes. When flowering is strongly displaced at higher altitudes, flower longevity at different altitudes could be similar because of convergence of temperatures at high and low altitudes. Permission to work on the three sites was granted by Ricardo Margulis, General Manager of the private Valle Nevado Ski resort and Humberto Gallardo, who manages private land below the ski complex. Sites were fenced to keep summer-grazing goats, cows and horses at bay. Slope and aspect were uniform within each site. LOW and MID were flat sites with no predominant exposition; HIGH faced west. MID turned out to be more protected from afternoon winds and was characterized by sandier soils that LOW.

**Table 1:** Site characteristics and potential flower longevity in *Rhodolirium montanum* at different altitudes and over two summer seasons.

Austral summer season	Site	Altitude (m a.s.l.)	Mean air temperature at 1.5 m a.g.l. (°C)	Mean air temperature at flower height in cm a.g.l. (°C)	Mean potential flower longevity in days (range)
**2013–2014 (Yr 1)**	LOW	2350	15.6	16.2	8.1^a^ (1–13)
	HIGH	3075	11.7	12.5	8.1^a^ (1–16)
**2014–2015 (Yr 2)**	LOW	2350	14.9	16.4	5.7^a^ (1–10)
	MID	2650	15.0	16.0	6.3^b^ (3–10)
	HIGH	3075	10.9	12.9	7.8^c^ (3–11)

Also shown are average air temperature at 1.5 m a.g.l. and at flower height a.g.l. over the days a flower remains open, averaged over all flowers on a site. Different letters indicate significant differences within years.

### Supplemental warming

We assessed whether flowers in *R*. *montanum* have the capacity to respond plastically to temperature in a supplemental warming experiment (cf. [44]) from 5 January to 15 February, 2011 on LOW. Twenty Open Top Chambers (OTCs) (details of model in [60]) were placed over patches of five or more genets of *R*. *montanum* while still in a vegetative state. Identical chambers produced a 2.8°C increase over ambient temperature in flower longevity work carried out by us on another alpine species in the same general study area [44]. Flowers on five or more genets in each of 20 hexagons equal in area to the six-sided OTCs served as controls. Before the control and treatment flowers opened, they were fitted with muslin pollinator exclosures mounted on a basal ring and supported by wooden stakes [44]. We purposely did not emasculate the flowers because emasculation is known to reduce flower longevity [31,61]. We monitored treatment and control plants daily registering the day of anthesis and senescence for all flowers that appeared in the controls and OTCs over the full flowering season. Flower longevity was the number of days a flower remained open.

### Altitudinal variation

To detect altitudinal trends and assess the contribution of temperature around flowers in shaping altitudinal variation in flower longevity we measured potential flower longevity in natural populations at different altitudes controlled for external pollination over a large part of the flowering season at each altitude. We cordoned off large areas of the populations on the three sites at the beginning of the flowering season then visited those areas on a daily basis, incorporating new previously marked flowers as they appeared until the flowering season was about to end. External pollination was controlled for by covering all flowers monitored for their longevity with the same transparent muslin tent-like exclosures used in the supplementary warming experiment. Again, we purposely did not emasculate flowers because emasculation can reduce flower longevity [31,61]. Data on flower longevity for LOW and HIGH was obtained over two consecutive austral summers (2013–2014 austral summer—HIGH: 23 December to 2 February, 2014; LOW: 23 December, 2013 to 1 February, 2014; 2014–2015 austral summer–HIGH: 31 December, 2014 to 20 January, 2015; MID: 2–24 January, 2015; LOW: 31 December to 23 January, 2015). As can be seen, flowering times at different altitudes strongly overlap. In Yr 1 we only considered the altitudinally extreme sites. However, as additional funds came available in the second year we were able to include the MID site. Inclusion of two summer seasons allowed us to obtain flower longevity records over a wide range of ambient temperatures to which flowers of *R*. *montanum* are exposed and detect inter-annual variation in flower longevity. The former was essential to provide sufficient temperature variation for statistical purposes. Total numbers of days of observation on flowers were 2013–14 (Yr 1): 32 (LOW) and 38 (HIGH); 2014–15 (Yr 2): 18 (LOW), 17 (MID) and 17 (HIGH). In Yr 1, a wetter year [62], flowering lasted considerably longer than in Yr 2.

Differences in flower size have been found to increase, decrease or have no effect on flower longevity [12,63,64]. Although we attempted to measure flower diameter to represent flower size on intact flowers in Yr 2, it was impossible to obtain an accurate measure of flower size because the tepals of *R*. *montanum* curve backwards as the day progresses. Under these circumstances flower measurements will only be valid if made at a standard time of development on all sites. This was out of the question in our study given the large number of flowers that had to be monitored on the three sites every day. Not being able to incorporate flower size into our analysis of altitudinal variation in flower longevity however was not a serious limitation. An analysis of the dry weights of 25 fresh haphazardly selected flowers (1 per genet, including the tepals and ovary) per site showed that while there was a significant site effect on flower mass (One-Way ANOVA: F_2,72_ = 3.549, P = 0.0339), there was no clear altitudinal trend (S1 Fig). Flowers on MID were slightly heavier and only significantly different with LOW (t_72_ = 2.658, P < 0.05); other differences were not significant. Thus, it is unlikely that flower size could explain the predicted altitudinal increase in flower longevity in *R*. *montanum* due to temperature.

Although *R*. *montanum* is described as non-autogamous [57], on-site observations and dry flowers recuperated late in the season from the natural populations, revealed a few large fruits containing varying numbers of good seed, along with a considerable number of swollen ovaries that appeared to be very early aborted fruits. The small fruits contained numerous transparent expanded ovules accompanied by a few opaque ovules that could have arisen from sporadic self-pollination followed by early abortion. Alternatively, they represent some form of parthenocarpy. Sporadic self-pollination is not impossible in pollinator-excluded flowers because pollen tends to become adhered to sides of the muslin tents as they cave in on windy days and from there could be transferred accidently to the stigmas. As self-pollination can hasten flower senescence [65], to avoid this unwanted source of variation in the natural populations, we finally decided to eliminate all flowers known to form mature and small aborted fruits prior to carrying out the statistical analyses. Sporadic low-level selfing could have occurred in the supplemental warming experiment, but is inconsequential because it would have affected both the controls and experimental plants to a similar degree. Unfortunately, not all flowers on HIGH could be recuperated at the fruiting stage for logistic reasons in Yr 1. We checked to see if this would have affected our results on HIGH by comparing the longevity of flowers that had accidently received some self-pollen and flowers that clearly had not in Yr 2, finding no significant difference (Welch´s t-test for unequal sample sizes: t = 0.186, P = 0.854). For the other sites we found a significant decrease in flower longevity for self-pollinated flowers on MID (t = 2.960, P = 0.008) and a non-significant difference for LOW (t = 0.960, P = 0.340). Therefore, not being able to revise all flowers on HIGH in Yr 1 was probably inconsequential. Overall, excluding flower losses to insect, occasional domestic animal, and wind damage, and the problematical flowers, we obtained flower longevity data for 1861 flowers.

### Temperature measurements

#### Supplemental warming

In order to determine the temperature increase afforded by the OTCs, we recorded temperature with hobos (Onset Computer Corp., Cape Cod, MA, USA) fitted with shades at 15 min intervals at a standard height of 15 cm above ground level (a.g.l.). One hobo was placed in each OTC and control patch, respectively. From the hobo recordings, we calculated the mean daily temperature for the exact dates over which each individual flower remained open, thus obtaining a tightly-controlled measure of the temperature environment experienced by each warmed and control flower.

#### Altitudinal variation

Eight or more hobos were located at 15 cm a.g.l. on each site over the two summers of observation in natural populations. Again, from these data we calculated the mean daily temperature for the exact dates over which each flower remained open to obtain a measure of an individual flower´s immediate temperature environment. The height above ground at which temperature sensors are placed is not trivial when flower height varies over the altitudinal gradient. Plant stature and flower height a.g.l. tend to become reduced at high altitude [66,67]. At the same time temperature increases as distance from the ground decreases [42,55]. As work progressed in Yr 1, notable differences in flower height along the altitudinal gradient became evident. Therefore, in Yr 2 we took steps to adjust the raw temperature data recorded at 15 cm a.g.l. to represent more realistic flower height on each site. First, we measured the orthogonal distance from the point of insertion of the flower on the peduncle to the ground for the flowers monitored for longevity on the three sites to obtain a standardized measure of flower height. Second, we developed vertical temperature profiles on each site by recording temperature at 5 cm, 10 cm, 15 cm and 20 cm a.g.l. with hobos placed in the center of each site for a period of 37 days. Using natural splines fitted to the resulting vertical profiles (S1 Table), we adjusted the original temperatures recorded at 15 cm a.g.l. for the dates each flower remained open by +0.5°C, +0.1°C and +0.7°C for, LOW, MID and HIGH flowers, respectively. Because flower height was only available for the second flowering season, we assumed that average flower height on each site was the same for the two flowering seasons. We also measured temperature at 1.5 m a.g.l. with one shaded hobo per site throughout the entire flower season for Yr 1 and Yr 2 (Table 1) in order to compare temperatures at standard meteorological height and flower height above ground level on each site.

### Statistical treatment

#### Supplemental warming

Flower longevity for the warmed and control plants was analyzed with a Linear Mixed model (LMM). Model validation was ratified with graphical analysis of scaled residuals [68]. For the LMM analysis, treatment was considered as a categorical factor (OTC or control) and genet (n = 85 for control; n = 99 for treatment) as a random factor nested in treatment. Hypothesis testing was with the F test based on Satterthwaite’s approximation of degrees of freedom for fixed effects and the likelihood ratio test for random effects.

#### Altitudinal variation

Flower longevity in natural populations was analyzed with a Linear Mixed Model (LMM). Model validation was ratified with graphical analysis of scaled residuals [68]. Mean temperature at flower height above ground was treated as a covariate and site (LOW, MID, and HIGH) and year (Yr1, Yr2) as categorical factors. Genet (n = 275 for LOW; n = 62 for MID; n = 302 for HIGH) was a random factor nested in site. For the LMMs we used the Bates et al. [69] *lme4* package and reported results according to the Bayesian Information Criterion (BIC) and the Akaike Information Criterion with correction for finite samples (AICc) [70]. Because flower height did not comply with the required assumptions of parametric models, we analyzed flower height with a k-sample non-parametric comparison (Kruskall-Wallis).

## Results

### Supplemental warming

**Fig 2:**
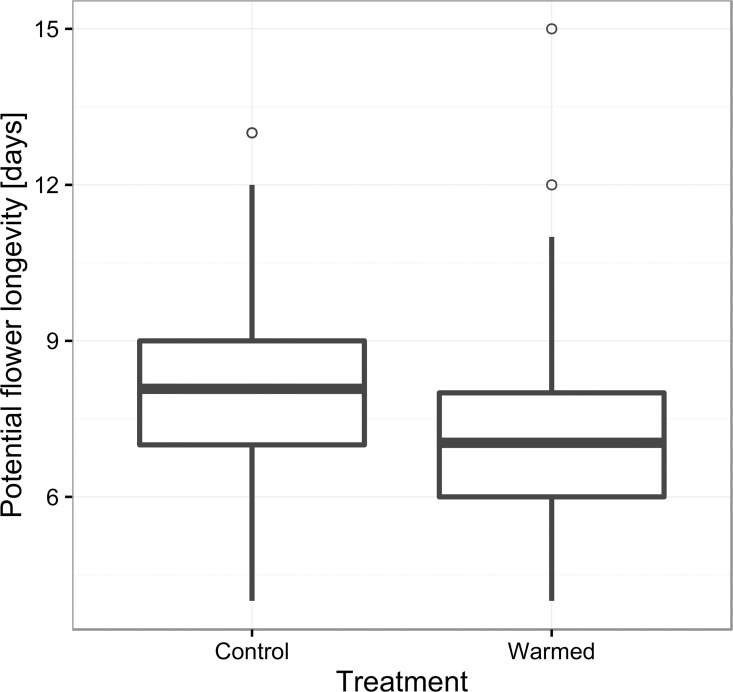
Modified boxplot for potential flower longevity in supplementally warmed and control flowers. The central line shows mean flower longevity.

Mean daily temperatures inside and outside the OTCs were 17.8 ± 0.05°C and 14.7 ± 0.04°C, respectively. Supplemental warming had a significant negative effect on flower longevity (Table 2, Fig 2). The effect of genet was also significant (Χ^2^ = 12.9; DF. = 1, P = 0.0003). The difference of 3.1°C in mean temperatures experienced by the control and warmed flowers led to an estimated decrease in flower longevity of 1.2 ± 0.2 days, providing a flower longevity rate of change of -0.387 days°C^-1^.

**Table 2:** Hypothesis testing for the effect of supplemental warming on potential flower longevity from the LMM.

Fixed effect	Estimate	Std. Error	Sum Sq	Mean Sq	Num. DF	Den. DF	F value	P
Intercept	7.0349	0.1484						
Treatment	1.1456	0.193	83.097	83.097	1	240.82	35.2259	<0.0001

### Altitudinal variation

**Fig 3:**
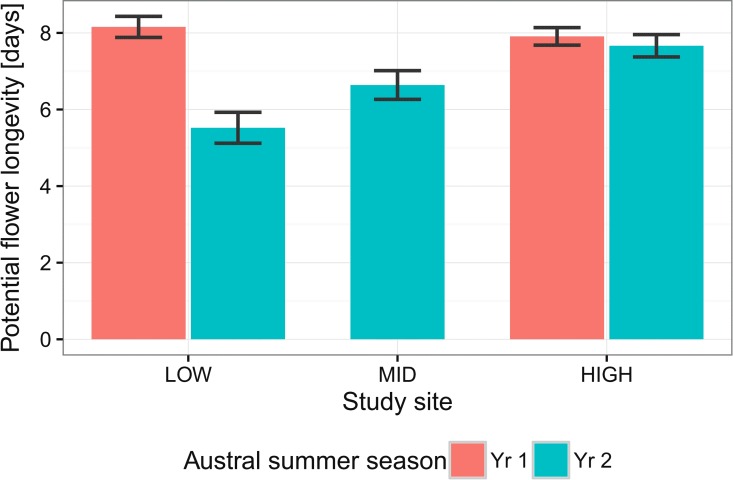
Mean potential flower longevity in *R*. *montanum* on different sites over the austral summers of 2013–2014 and 2014–2015. Error bars indicate the 95% confidence interval for the mean.

Temperatures around flowers in natural populations were 3.7°C (Yr 1) and 3.5°C (Yr 2) lower on HIGH in comparison with LOW (Table 1). The corresponding temperatures differences at 1.5 m a.g.l. were higher for both summers (Table 1). Notably, temperature at flower height was as much as 2.0°C warmer than at 1.5 m a.g.l., the equivalent of a difference of well over 300 m of altitude on a typical altitudinal gradient. Much variation in potential flower longevity was found on each site (Table 1), covering a total range for all sites from one to sixteen days. Flower longevity across sites and years averaged 5.7 to 8.1 days (Table 1). In Yr 1 mean flower longevity was the same on HIGH and LOW (Fig 3) and not surprisingly, the contrast of marginal means with site as a fixed factor did not reach statistical significance (P = 0.254). For Yr 2, significant differences were found among all pairs of sites (HIGH vs MID, P < 0.0001; MID vs LOW, P < 0.001; LOW vs HIGH, P < 0.0001) there being a progressive increase in flower longevity with increasing altitude (Fig 3). According to both the BIC and AICc criteria the site x year model best explained natural flower longevity variation across the altitudinal range (Table 3). The second best model was the interaction temperature x year, but this had low explanatory value. Temperature alone had even lower explanatory power, as did the interaction temperature x site. Thus there is no evidence that natural variation in potential flower longevity across the altitudinal gradient is a direct product of temperature around flowers.

**Table 3:** LMM model comparisons for variation in potential flower longevity in natural populations of *R*. *montanum* along an altitudinal gradient.

Fixed factors for candidate models	D.f.	BIC	AICc	BIC weight	AICc weight
Site x Year	7	7177.5	7138.8	0.998	1
Temperature x Year	6	7189.5	7156.4	0.002	0
Year	4	7206.7	7178.9	0.000	0
Site	6	7213.8	7225.3	0.000	0
Temperature x Site	8	7215.1	7235.3	0.000	0
Temperature	4	7262.1	7240.0	0.000	0

**Fig 4:**
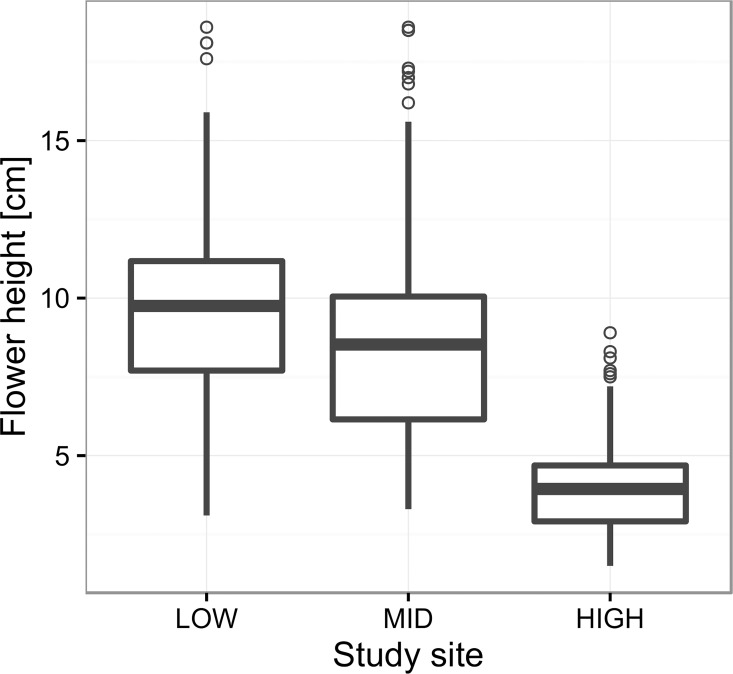
Flower height in *Rhodolirium montanum* at three altitudes in the central Chilean Andes. Modified boxplot where the central thick line indicates mean flower height.

Finally, site had a highly significant effect on flower height above ground level (X^2^ = 344.16, df = 2, p <0.0001). Flower height decreased strongly with increasing altitude from an average of 10.3 ± 0.36 cm on LOW to 3.9 ± 0.16 on HIGH; differences were significant between all pairs of sites (Fig 4).

## Discussion

The altitudinal trends in potential flower longevity in *R*. *montanu*m were surprisingly different over the two years. While flower longevity was more prolonged at the higher altitudes in one year, there was no altitudinal increase in another year. Flower longevity among years converged in the upper part of the altitudinal range, varying little between years. To the contrary, in spite of quite similar temperatures around flowers on the same site among years, significant inter-annual variation was seen in the lower part of the altitudinal range. Inter-annual variation on the same site points to a high degree of flower longevity lability in *R*. *montanum*. Similar inter-annual variation in potential flower longevity has been documented for *Gentiana straminea* on the Qinghai-Tibetan Plateau [36]. The supplemental warming experiment verified the underlying supposition underpinning our main hypothesis: i.e. flowers of *R*. *montanum* can respond plastically by changing their longevities in accordance with ambient temperature. However, although we obtained good experimental evidence that *R*. *montanum* flowers can respond plastically to temperature, and flowers stayed open for longer with increasing altitude in one year, the LMMs failed to support the hypothesis that the temperature environment around flowers explains site variation in potential flower longevity along *R*. *montanum´s* altitudinal range.

What then drives natural variation in potential flower longevity in *R*. *montanum* and what is responsible for the strikingly different altitudinal patterns among years? The highly ranked candidate site x year model signifies that flower longevity changes on sites among years and leads to the following inferences. Shorter-lived flowers on LOW in Yr 2 correspond to a year in which the spring and summer were unusually dry [62]. Indeed, unlike in the previous summer (Yr 1), no rain was received on LOW over *R*. *montanum´s* entire flowering season. Drought stress is known to induce ethylene synthesis and provoke flower senescence in some cultivated plants [71] and water availabilty to affect flower size [72,73]. In some species drought-induced senescence is associated with an elevated ABA level [33]. Recently soil water status has been experimentally shown to reduce flower longevity in lowland *Collinsia heterophylla* [74]. It would have been useful to know the extent to which dry and fresh flower weights in *R*. *montanum* flowers differ over our altitudinal gradient. Water content in flowers is expected to be lower at lower altitudes in the central Chilean Andes.

Differences in soil moisture could also explain the fact that flower longevity was more prolonged on MID than on LOW (Table 1) in Yr 2 in spite of the fact that mean daily temperatures around the sets of flowers that were monitored were not very different on these two sites due to topographic effects and soil properties and possibly the reduced flower height on MID compared to LOW (see later discussion on flower heights). Data available for Yr 2 on our sites showed that soil volumetric water content descended from 38.8% on HIGH to 5.0% on LOW (S2 Table). Lower water availability and higher temperatures at lower elevations would increase floral physiological costs, which is turn would diminish the chances of increasing reproductive success due to resource limitation for producing seeds and fruits [23]. Indeed, the capacity for rapid plastic adjustment of flower longevity under drought might have been selected in *R*. *montanum* as a means for not compromising fruit and seed set in dry years. On a larger spatial scale, drier soils might explain why flower longevity in the central Chilean alpine with a mediterranean-climate influence is generally less prolonged than in wetter mountain systems in New Zealand and the Swiss Alps [12,75], although given that the available data is for open-pollinated flower longevity, higher pollination rates in the central Chilean Andes cannot be discarded. Also, although our warming experiment was conducted in a more normal summer in terms of precipitation, some soil drying under higher temperature might have contributed to the reduction in flower longevity obtained with passive warming. Sierra-Almeida & Cavieres [76] documented significant soil drying inside OTCs several months after they had been placed over plants. Assessing the relative importance of soil moisture, the direct effect of temperature as it affects flower respiration rates and rate of flower development [52,53,77], and the indirect effect of temperature as it affects water loss in flowers is critical for our understanding of the drivers of potential flower longevity along altitudinal gradients, especially in dry alpine areas.

An experimental temperature increase of 3.1°C in the OTCs produced an average 1.2 days decrease in flower longevity, yet flower longevity in natural populations was identical on LOW and HIGH in Yr 1 in spite of an even greater temperature difference between those two sites (3.5°C). That the models failed to detect that temperature contributes to site variation in potential flower longevity over the altitudinal gradient, as we had hypothesized, thus is not altogether surprising. Two explanations may be offered for the similar longevities at the extremes of *R*. *montanum´s* altitudinal distribution in a more normal year. As *R*. *montanum* colonized into high altitudes, perhaps its flowers became acclimated to cooler temperatures. Respiration rates typically increase in response to short-term increases in ambient temperature and indeed flower respiration rates are known to increase with flower tissue temperature under laboratory conditions [52]. Although cool temperatures associated with lower respiration rates prolong flower lifespans in cut flowers [53,54], it is known that longer term responses to temperature in other plant organs may differ from short-term responses since respiration acclimates to the thermal environment [78–80]. Flower respiration rates have been measured for some alpine species [20,81], but research on acclimation and its effect on rates of flower development, and ultimately potential flower longevity, is lacking. Second, counterintuitively, there could have been selection to *reduce* flower longevity in the upper part of *R*. *montanum´* range. It seems unlikely that selection would have increased flower longevity on the lower part of the altitudinal range to match that on the upper part of the latitudinal range, which would be an alternative possibility. Fruit development and seed ripening in general are strongly temperature-dependent in the alpine [82,83]. Failure to ripen seed in unseasonably cold and late summer years is recognized as a recurrent bottleneck in alpine plants [45] and will be more strongly manifest in the upper parts of alpine species´ altitudinal ranges. Selection to reduce potential flower longevity would tend to free up resources for fruit and seed production. Needless to say, neither the physiological nor the evolutionary explanation for the lack of an altitudinal increase in potential flower longevity in a more normal year signifies that potential flower longevity in *R*. *montanum* is not temperature sensitive, only that the relationship will not be easily detectable as a result of other effects of the kinds just outlined.

An interesting result coming out of our study was the finding that flower height in *R*. *montanum* declined notably as altitude increased. Flowers on the highest site were disposed fully 6.4 cm closer to the ground than on the lowest site. This difference translates to an overall decrease in flower height of 0.88 cm per 100 m increase in altitude. An altitudinal reduction in flower or inflorescence height above ground has been reported in other alpine species [7,58] and is likely to be globally common given that in general plant stature decreases with altitude in the alpine life-zone [45,66,67,84].

In species in which flower longevity is temperature sensitive, as is the case in *R*. *montanum*, a reduction in flower height could lead to a counter altitudinal tendency consisting of a plastic decrease in flower longevity because as flowers become disposed closer to the ground they are likely to encounter increasingly higher temperatures (cf. [42,55]). Precisely due to differences in flower height across the altitudinal gradient, the temperature difference at flower height between LOW and HIGH was seen to be smaller than for temperature measured at 1.5 m a.g.l. Although a decrease in flower height across the altitudinal gradient might have contributed to the similar flower longevities at the two altitudinal extremes in Yr 1, it is clearly not the only factor, as a large difference in temperature around flowers still remained between the two altitudinal extremes (3.5°C). In any case, a reduction in flower height with increasing altitude perhaps is a better overall strategy for *R*. *montanum* than maintaining taller, longer-lived flowers subject to cooler conditions. Shorter flowers placed in a warmer thermal layer would tend to favor higher pollination rates because of the clear preference of pollinators for warmer temperatures at high altitudes [1,41], resulting in more abundant and earlier pollination in a flower. Earlier pollination, moreover, would add additional days for fruit and seed maturation. The fitness trade-off between maintaining taller, more longer-lived flowers versus reducing flower height and having less prolonged flower longevity both as a local plastic effect and possibly due to selection, is a theme that warrants detailed study along thermal gradients.

Finally the literature reveals a number of cases where flower longevity has been studied in pollinator-excluded flowers over an altitudinal gradient that show no or very little altitudinal increase in potential flower longevity [17,36,37]. Including our results in *R*. *montanum* for a more normal year, increases that number. Additionally, Steinacher & Wagner [18] found no altitudinal increase in potential flower longevity for a comparison of different species drawn from the alpine and nival-subnival zones in the European Alps. Overall, although the sample size is still very small, there seems to be relatively little evidence for an altitudinal increase in potential flower longevity measured in pollinator-excluded flowers in comparison for flower longevity measured in open-pollinated flowers. Whether previously reported cases are true cases of an increase in potential flower longevity versus a consequence of a depression in flower longevity at lower altitudes, as we found in Yr 2 in *R*. *montanum*, remains to be seen. Altitudinal trends in potential flower longevity where plastic effects, physiological adjustment and selection on flower longevity are likely to be combined, clearly are more complex than altitudinal trends in longevity in open-pollinated flowers reflecting more a snap-shot of differences in levels of pollinator activity on flowers at different altitudes.

## Conclusions

In *R*. *montanum* we found good experimental evidence for a rapid plastic response of flowers to ambient temperature consisting of a shortening of potential flower longevity under higher temperatures, but failed to find evidence that a decline in temperature around flowers contributes to an altitudinal increase in potential flower longevity in natural populations. In a wetter year no altitudinal increase in potential flower longevity was evident. However, severe soil drying in a drier year was associated with a significant depression in flower longevity in the lower part of *R*. *montanum´s* altitudinal range. Potential flower longevity in *R*. *montanum* thus is a highly plastic floral trait affected by abiotic factors. The take home message coming out of this study is that what might be interpreted as an altitudinal increase in potential flower longevity in the alpine from the point of view of providing more time for pollination in flowers, could often be caused, at least partially, by a depression in flower longevity at lower altitudes due to abiotic effects. Overall, compared with open-pollination flower longevity, the emerging picture suggests an increase in flower longevity along the altitudinal gradient is less common than for potential flower longevity. Finally, we stress that our results do not negate that selection has occurred on potential flower longevity in *R*. *montanum* and suggested where this might have occurred. Irrespective of whether selection to prolong (or shorten) flower longevity has taken place in some high altitude species or not, we predict plastic effects on flower longevity such as those found here, will be commonplace in alpine species growing in dry climates. Such environmentally-produced lability in flower longevity is likely to push alpine species towards shorter flower life spans under climate change in areas like the central Chile alpine where instrumental records show temperature has increased over the past three decades [85] and precipitation is expected to decline [86]. As individualistic responses of pollinators to climate warming are expected, the impact of shorter-lived flowers under climate change on plant fitness is unpredictable.

## Supporting Information

S1 Fig Flower dry weights for *Rhodolirium montanum* on the LOW, MID and HIGH sites. A modified boxplot where the central thick line indicates the mean flower dry weight by study site.
(TIF)

S1 Table Estimates of intercepts and spline coefficients for reduction in temperature with increasing height above ground on three Andean sites based on temperature recorded with hobos at 5, 10. 15 and 20 cm a.g.l.
(DOCX)

S2 Table Mean TDR measurements of soil moisture on LOW, MID and HIGH for selected dates during the 2014–2015 flowering season of *R*. *montanum*. (DOCX)
