# Supplementary material for: Plastic Responses Contribute to Explaining Altitudinal and Temporal Variation in Potential Flower Longevity in High Andean Rhodolirion montanum
Source: PLoS One. 2016 Nov 18;11(11):e0166350. doi: 10.1371/journal.pone.0166350 (PMC5115873; doi:10.1371/journal.pone.0166350)
Supplement: S1 Table — 15 and 20 cm a.g.l. (DOCX) [file pone.0166350.s002.docx]

**S1 Table. Estimates of intercepts and spline coefficients for reduction in temperature with increasing height above ground on three Andean sites based on temperature recorded at 5, 10. 15 and 20 cm a.g.l.**

| **Site** | **Intercept** | **Height** | **Height (≥ 10cm)** | **Height (≥ 15 cm)** |
| --- | --- | --- | --- | --- |
| LOW | 17.156 | -1.223 | -2.286 | -1.132 |
| MID | 17.2794 | -0.4644 | -2.1787 | -0.2696 |
| HIGH | 12.7550 | -0.2720 | -1.2915 | -0.3159 |
