# Supplementary material for: Plastic Responses Contribute to Explaining Altitudinal and Temporal Variation in Potential Flower Longevity in High Andean Rhodolirion montanum
Source: PLoS One. 2016 Nov 18;11(11):e0166350. doi: 10.1371/journal.pone.0166350 (PMC5115873; doi:10.1371/journal.pone.0166350)
Supplement: S2 Table — (DOCX) [file pone.0166350.s003.docx]

**S2 Table. Mean TDR measurements of soil moisture on LOW, MID and HIGH for selected dates during the 2014-2015 flowering season of *R. montanum*.**

| **Study site** | **N** | **Mean volumetric water content (%)** | **SEM** | **SD** |
| --- | --- | --- | --- | --- |
| HIGH | 6 | 27.4 | 3.3 | 8.2 |
| MID | 6 | 28.8 | 2.8 | 6.8 |
| LOW | 21 | 5.7 | 0.4 | 1.6 |

N: Sample size; SEM: Standard error of the mean; SE: Standard Deviation
